# Correlates of normal and decreased HDL cholesterol levels in type 2 diabetes: a cohort-based cross-sectional study

**DOI:** 10.1186/s12944-024-02010-6

**Published:** 2024-01-19

**Authors:** Fatemeh Mohammadi, Amirhossein Yadegar, Soghra Rabizadeh, Aryan Ayati, Seyed Arsalan Seyedi, Seyed Ali Nabipoorashrafi, Alireza Esteghamati, Manouchehr Nakhjavani

**Affiliations:** 1 Endocrinology and Metabolism Research Center (EMRC), Vali-Asr Hospital, Tehran University of Medical Sciences, Tehran, Iran; 2 Research Center for Advanced Technologies in Cardiovascular Medicine, Tehran Heart Center, Tehran University of Medical Sciences, Tehran, Iran

**Keywords:** High-density lipoprotein, Triglyceride, Lipid profile, Restricted cubic spline, type 2 diabetes

## Abstract

**Background:**

The literature describes an inverse association between the values of triglyceride (TG) and high-density lipoprotein cholesterol (HDL-C). This survey was designed to exhibit the features of people with type 2 diabetes (T2D) who display this inverse association and identify potential contributing factors to having normal HDL-C values.

**Methods:**

A total of 6127 persons with T2D were assigned to the present survey. Demographic features and clinical status data were compared between subjects with a substantial inverse association of TG and HDL-C and those without. Logistic regressions were performed to ascertain the role of different factors related to normal HDL-C. Moreover, the restricted cubic spline (RCS) functions were conducted to scrutinize the underlying relationships between the studied variables and low HDL-C levels.

**Results:**

Patients with high TG (150 ≤ TG < 400) compared to patients with normal TG (TG < 150) were less likely to have normal HDL-C. Younger age, narrow hip, lower levels of blood pressure, two-hour postprandial glucose (2hPP), fasting blood sugar (FBS), hemoglobinA1C (HbA1C), low-density lipoprotein cholesterol (LDL-C), total cholesterol, and non-HDL-C, higher atherogenic index of plasma (AIP), and TG/HDL-C ratio correlate with an inverse connection between the values of HDL-C and TG (all *P* < 0.05). Age greater than 65 years (odds ratio (OR) 1.260, 95% confidence intervals (CI) 1.124-1.413) had a positive association, whereas female sex (OR 0.467, CI 0.416-0.523) , 25 kg/m2 < body mass index (BMI) (OR 0.786, CI 0.691-0.894), and higher serum creatinine levels (OR 0.481, CI 0.372-0.621) had an inverse association with having normal HDL-C.

**Conclusions:**

Patients with an inverse connection between TG and HDL-C values had considerably different anthropometric features, lipid profiles, and glucose indices compared to those without this relationship. Furthermore, patients who aged less than 65 years, had female gender, BMI more than 25 kg/m2, and higher serum creatinine levels were less likely to exhibit normal HDL-C levels.

## Introduction

The overall prevalence of dyslipidemia is approximately 25·5%, 27%, 30.2–47.7%, 53%, and 80%, while the prevalence of low high-density lipoprotein cholesterol (HDL-C) is as high as 37.4%, 35.4%, 22.9–72.0%, 23%, and 69.2% within the general population in Africa, Europe, the Asia–Pacific region, the United States, and Iran, respectively [1–5]. Previous studies have consistently delineated that elevated concentrations of HDL cholesterol are an established inverse predictor of atherosclerotic cardiovascular disease [6]. A decreased concentration of HDL-C was the primary lipid abnormality reported in approximately half of the persons with known coronary heart disease [7]. Only a 1 mg per deciliter (0.03 mmol per liter) elevation in HDL-C levels can lower the risk of future cardiovascular events by 2–3% [8]. Moreover, these heterogeneous lipoproteins have other protective functions, such as antioxidative, anti-inflammatory, antiapoptotic, and immune-modulating effects [9]. Low HDL cholesterol amounts have been documented in various malignancies, such as prostate, lung, and breast [10]. Each drop of 1 mg/ dL HDL-C level could be associated with an increase of 14% in cancer risk [9].

Despite profound advances in pharmacologic and interventional care, increasing HDL-C amounts has been documented to be a daunting task due to its controversial response to available drugs [11]. The impact of statins on HDL-C is controversial; analyses of data have added support to the proposition that statins may correspond to only a 5–10% increase or no changes in HDL-C levels [7, 12, 13]. Dietary therapies have also not yielded an appreciable elevation in HDL-C values [14]. Following the release of apolipoprotein A-I from the liver, the life cycle of HDL emerges. The binding of apolipoprotein A-I to circulating phospholipids and cholesterol participates in the development of discoid lipid-poor HDL, which provokes cholesterol efflux and, subsequently, cholesterol accumulation in the core of these particles. Then, cholesterol transfers into the liver via two mechanisms: a direct pathway through scavenger-receptor B-I and an indirect pathway through low-density lipoprotein (LDL) or very low-density lipoprotein (VLDL) particles, which is mediated by cholesterol ester transfer protein (CETP) [11]. As triglyceride (TG) increases, the core and surface domains of HDL-C and LDL-C reform. Therefore, both high and low-density lipoproteins become denser and smaller due to the replacement of cholesterol ester with TG. As a result, plasma concentrations of TG have a regulatory role in the structure of lipoproteins [14, 15]. Earlier studies showed a strong association between high TG values and decreased HDL-C amounts [15]. However, the interrelationship between TG and HDL cholesterol values and contributing factors need further investigation.

Since current treatment schemes are unlikely to raise HDL-C satisfactorily, further therapeutic strategies that target HDL-C metabolism step into the spotlight [11]. Therefore, the discovery of factors influencing HDL may assist in the development of more effective treatment methods. In the previous studies, TG was presented as a factor that could have a reciprocal effect on HDL-C levels. However, it should be noted that not all individuals exhibit a connection between their HDL-C and TG concentrations [14, 15]. Additionally, limited data has been published regarding the inverse connection between HDL-C and TG in populations with type 2 diabetes (T2D). The objective of the current survey was to further define the characteristics of individuals who were more likely to display this inverse relationship. The present study investigated the interrelationship between TG and the values of HDL-C in T2D individuals with concurrent decreased HDL-C and elevated TG and persons with concurrent raised TG and normal HDL-C and variables influencing this connection. Considering the lack of promising shifts in the trend of HDL-C amounts [16–18], exploring additional variables linked to HDL-C levels could assist in optimizing patient care. Hence, this report concentrated on the attributable elements that increased the likelihood of having normal HDL-C. These factors may offer insight into the pathogenesis of low HDL levels and additional therapeutic avenues in the future.

## Method and material

### Study design and population

Retrospective data on 7391 consecutive individuals with known T2D recruited at a university hospital from 2016 through 2021 for follow-up was obtained. The inclusion criteria were defined as having T2D, being at least 18 years of age, and having consent to engage in the survey. The exclusion parameters were described as a positive history of thyroid disease, dialysis, chronic renal disease (CKD) with an estimated glomerular filtration rate (eGFR) < 30, malignancy, liver cirrhosis, pregnancy, smoking, taking any antiplatelet therapy, oral contraceptive drugs, or antioxidant supplements, and patients who were not receiving statins. As stated by the American Diabetes Association (ADA) to preserve LDL concentrations of less than 70 mg/dL in diabetic subjects, about 95% of recruited patients were prescribed statins and those not receiving statins were excluded. As a result, 6127 patients were eligible for enrollment. A high proportion of the target population was homogenous, attained a middle to high school level of education, and possessed a middle socioeconomic status. In addition, most of them had access to medical care and insurance services. The studied subjects suffered from T2D and had their regular daily activity and diet, and they were not justified to have excessive physical activity. The regular diet in Iran consists of 450 g of carbohydrate per day (more than 60% of all consumed calories), followed by 12.3 g trans-fat in the daily diet, accounting for 4.2% of total calorie intake [19, 20]. The participants in this study also followed a regular diet. Moreover, the studied population had their habitual physical activity. According to the national report, around 40% of the adult population in Iran is classified under the low physical activity category. Most of the physical activity was attributed to work (71%), 20% to commuting, and 9% to recreation. Around 15% of Iranian adults are not physically active [21]. Patients were receiving oral anti-diabetic agents (OADs), insulin, or their combination. Written informed consent was received from all studied populations. All research was in full compliance with the Declaration of Helsinki. The protocol of the study was approved by the Tehran University of Medical Sciences’ local ethics committee.

### Data collection

The following variables were established for each research participant: sociodemographic and anthropometric characteristics, as well as a blood sample. A standard questionnaire was administered to record sociodemographic features, including age, sex, and diabetic duration. Well-trained investigators carried out anthropometric measurements to record blood pressure, hip and waist circumference, weight, and height. A portable stadiometer and a calibrated balance beam scale were utilized to assess height and weight in the upright position, respectively. Following the Quetelet equation, the division of weight (kg) by the square of height (^m2^) was used for body mass index (BMI) calculation. Waist circumference (WC) was evaluated at halfway of the distance between the inferior margin of the rib cage and the iliac crest [22].

Meanwhile, the widest part of the buttock was calculated as the hip circumference. The waist-to-hip ratio (WHR) was computed by dividing WC by hip circumference. All measurements were accomplished with an accuracy of 0.1 cm. Blood pressure (systolic and diastolic) was documented using a calibrated mercury sphygmomanometer (Reishter, Germany) on the right arm while seated and experiencing a five-minute resting interval. Systolic blood pressure (SBP) and diastolic blood pressure (DBP) were obtained as the average of two assessments with a ten-minute interval, in which the participants were requested to sit and place their right hand at the level of their heart with the palm in the upward-facing position. The patients were asked to have their routine dietary habits during the week prior to study commencement. Following an overnight fasting period of 12 h, venous blood was gathered from each subject at 8 AM. The blood samples were drawn into ethylenediaminetetraacetic acid-containing (1.5 mg/ml) tubes and kept on ice until centrifugation. During a maximum period of 30 min, plasma was extracted using centrifugation at 3000 rpm and 4 degrees Celsius for 15 min. Serum lipid concentrations, including cholesterol, HDL, LDL, and TG, were assessed through direct enzymatic colorimetry utilizing a Technicon RA-analyzer (Pars Azmoon, Karaj, Iran). To estimate non-HDL cholesterol, HDL-C was subtracted from total cholesterol. The subsequent equation (log (TG/HDL-C)) was applied to compute the atherogenic index of plasma (AIP). The two following formulas were utilized to calculate the visceral adiposity index (VAI): (WC(cm)/(36,58 + (BMI *1.89) *(TG/0.81) *(1.52/HDL) for women and (WC(cm)/(39,68 + (1.88*BMI) *(TG/1.03) *(1.31/HDL) for men [23]. eGFR was computed via the Chronic Kidney Disease Epidemiology Collaboration (CKD-EPI) model [24]. Enzymatic calorimetric methods by the glucose oxidase test were utilized to evaluate two-hour postprandial glucose (2hPP) and fasting blood sugar (FBS). High-performance liquid chromatography (HPLC) (A1C, DS5 Pink kit; Drew, Marseille, France) was implemented to measure hemoglobin A1c (HbA1c) levels.

### Definitions

Diabetes diagnosis was made based on the ADA criteria [25]. Dyslipidemia was determined using AHA/ACC (The American Heart Association/The American College of Cardiology) and NCEP ATP III (National Cholesterol Education Program-Adult Treatment Panel III) recommendations [26, 27]. The guidelines consider low HDL cholesterol (< 50 mg/ dL in women and < 40 mg/ dL in men), high LDL-C (≥ 70 mg/ dL), high non-HDL cholesterol (≥ 130 mg/ dL), high total cholesterol (≥ 200 mg/ dL), high TG (≥ 150 mg/dL), and elevated AIP (> 0.24) as dyslipidemia patterns [28].

### Statistical analysis

All statistical analyses were performed using IBM SPSS software version 24.0 (SPSS Inc., Chicago, Illinois, USA) and STATA software version 14.0 (StataCorp LLC.). Continuous data were manifested as the mean ± standard deviation (SD). Categorical variables were represented as percentages and numbers. The baseline properties of the studied sample were compared using ANOVA and LSD tests. Statistical analyses comprised two basic steps. The first one was calculating the significance of the association between HDL and TG using a correlation coefficient. All patients were classified into different subgroups based on sex, HDL-C, and TG levels at this stage. Then, the group with a substantial association between HDL cholesterol and TG and the group with no significant connection were matched by their gender, TG levels, and duration of diabetes. Chi-square and an independent t test were performed to analyze between-group comparisons as indicated. Next, the strength of the correlation between the values of TG and HDL cholesterol was assumed by crude odds ratio and adjusted odds ratio. The adjustment was applied for age group (age cutoff: 65 years), sex, duration of diabetes group (duration of diabetes cutoff: 5 years), BMI group (BMI cutoff: 25 kg/m^2^), SBP, DBP, WHR, HbA1C, and creatinine. Moreover, a restricted cubic spline (RCS) was utilized with four knots centered at the 5th, 35th, 65th, and 95th percentiles of TG distribution to model the potential connection between the odds of having decreased HDL-C and the values of TG. Additionally, the odds of different demographic and clinical variables in normal HDL-C levels were investigated using univariate and multivariate binary logistics. The subsequent variables were entered in the multivariate logistic regression analyses: age group, gender, BMI group, HbA1c group, SBP, and creatinine. A two-sided *P* value of less than 0.05 was recognized as statistically significant.

## Results

### Baseline characteristics

A total of 6127 persons with T2D aged 57.72 ± 10.41 years were assigned to this study. Women constituted approximately 56.6% of the entire cohort of patients. Diabetes was recorded for an average of 9.61 ± 7.65 years among the studied patients. BMI (kg/m^2^) and WHR had mean (± SD) values of 28.85 ± 4.66 and 0.93 ± 0.05, respectively. Approximately 42.4% of participants were receiving antihypertensive drugs. The mean (± SD) values of SBP and DBP (mmHg) were 130.46 ± 17.76 and 78.87 ± 9.33, respectively. The mean (± SD) levels of HDL cholesterol (mg/ dL), LDL cholesterol (mg/ dL), and total cholesterol (mg/ dL) were 44.85 ± 11.00 (41.65 ± 9.89 in men, 47.30 ± 11.18 in women), 100.64 ± 34.56, 178.57 ± 44.57, respectively. The median level of TG was 150.00, with a range of 34.0 to 960.0. A total of 72.03% of the studied population were receiving atorvastatin, followed by rosuvastatin (27.97%). All participants were taking antidiabetic agents, including OAD (76.4%), insulin (14.7%), and their combination (8.7%). Despite diabetes medication use, the median HbA1c was 7.60 (range: 4.0%-15.0%), and the means (± SD) of FBS (mg/ dL) and 2hPP (mg/ dL) were 165.29 ± 60.58 and 228.80 ± 90.34, respectively. Creatinine (mg/ dL) and eGFR (ml/min/1.73 m^2^) had mean (± SD) values of 0.99 ± 0.22 and 83.11 ± 24.61, respectively. (Table 1).

**Table 1:** Characteristics of the patients at baseline

variable				Total (6127)	Normal TG (TG < 150) (3167)	High TG (150 ≤ TG < 400)(2960)
Age (yr)				57.72 ± 10.41^#^	58.85 ± 10.46	56.88 ± 10.19^a^
Female sex (%)				56.6	53.8	60.2
Duration of diabetes (yr)				9.61 ± 7.65	9.74 ± 7.92	9.52 ± 7.39
History of hypertension (%)				43	42	44.4
Weight (kg)				76.00 ± 12.93	74.51 ± 12.73	77.39 ± 12.88^a^
Height (cm)				162.42 ± 9.28	162.77 ± 9.30	161.92 ± 9.16^a^
BMI (kg/m^2)				28.85 ± 4.66	28.18 ± 4.72	29.54 ± 4.53^a^
Waist circumference (cm)				97.83 ± 10.09	96.51 ± 10.17	99.12 ± 9.79^a^
Hip circumference (cm)				105.42 ± 8.57	104.40 ± 8.49	106.43 ± 8.61^a^
WHR				0.94 ± 0.06	0.93 ± 0.06	0.94 ± 0.06^a^
Blood pressure (mm Hg)	Systolic			130.46 ± 17.76	129.75 ± 17.64	131.18 ± 17.82^a^
	Diastolic			78.87 ± 9.33	78.07 ± 9.23	79.63 ± 9.31^a^
On anti-hypertensive medications (%)				42.4	41.6	43.7
HbA1c (%)	Mean			7.83 ± 1.70	7.64 ± 1.60	8.00 ± 1.76^a^
	Median (range)			7.60 (4.0–15.0)	7.40 (4.0–14,7)	7.80 (4.0–15.0)
FBS (mg/ dL)				165.29 ± 60.58	157.58 ± 58.87	171.63 ± 60.51^a^
2hPP (mg/ dL)				228.80 ± 90.34	218.45 ± 86.84	237.12 ± 91.83^a^
Cholesterol (mg/ dL)	Total			178.57 ± 44.57	162.84 ± 37.64	191.83 ± 44.09^a^
	LDL-C			100.64 ± 34.56	93.87 ± 31.08	107.00 ± 36.46^a^
	Non-HDL			133.7 ± 42.9	116.28 ± 34.66	148.30 ± 41.73^a^
	HDL-C		Total	44.85 ± 11.00	46.56 ± 11.27	43.53 ± 10.38^a^
			Women	47.30 ± 11.18	49.44 ± 11.48	45.76 ± 10.48
			Men	41.65 ± 9.89	43.20 ± 10.02	40.16 ± 9.27
TG	Mean (mg/ dL)			173.46 ± 99.37	106.89 ± 25.39	219.02 ± 58.38^a^
	Median (mg/ dL) (range)			147.00 (34.0–398.0)	108.00 (34.0–149.0)	202.50 (150.0–398.0)
TG/HDL ratio				4.22 ± 3.06	2.44 ± 0.88	5.36 ± 2.11^a^
VAI				19.71 ± 3.89	19.22 ± 3.85	20.12 ± 3.86^a^
AIP				1.2 ± .6	0.82 ± 0.37	1.61 ± 0.36^a^
Creatinine (mg/ dL)				0.99 ± 0.22	0.99 ± 0.22	1.00 ± 0.22
Urea (mg/ dL)				31.78 ± 12.24	31.69 ± 12.47	31.87 ± 11.62
GFR (ml/min/1.73 m^2^)				83.11 ± 24.61	81.03 ± 24.12	84.70 ± 24.69^a^
Medications						
OAD (%)				76.49	76.41	76.63
Insulin + OAD (%)				8.74	8.80	8.64
Insulin (%)				14.77	14.79	14.73
Lipid lowering drug		Atorvastatin (%)		72.03	72.30	71.62
		Rosuvastatin (%)		27.97	27.70	28.38

*BMI* body mass index, *HbA1C* hemoglobin A1C, *FBS* fasting blood sugar, *2hPP* two-hour postprandial glucose, *WHR* waist-to-hip ratio, *GFR* glomerular filtration rate, *TG* triglyceride, *LDL-C* low-density lipoprotein cholesterol, *HDL-C* high-density lipoprotein cholesterol, *AIP* atherogenic index of plasma, *VAI* visceral adiposity index, *OAD* Oral anti-diabetic agents

^#^Data are mean ± SD

^a^*P*value < 0.05 vs normal TG

Demographic features of patients in two subgroups stratified by TG levels (defined as normal TG (TG < 150, 49.9% or *N* = 3167) and high TG (150 ≤ TG < 400, 46.7% or *N* = 2960) were also compared. A substantial difference was observed in age, hip and waist circumference, WHR, BMI, weight, height, SBP, DBP, HbA1C, FBS, 2hPP, lipid profile and parameters, and eGFR between the two classifications. All clinical, anthropometric, demographic, and laboratory details of the studied sample in each subgroup are delineated in Table 1.

### Correlation between concurrent values of HDL cholesterol and TG

**Fig. 1:**
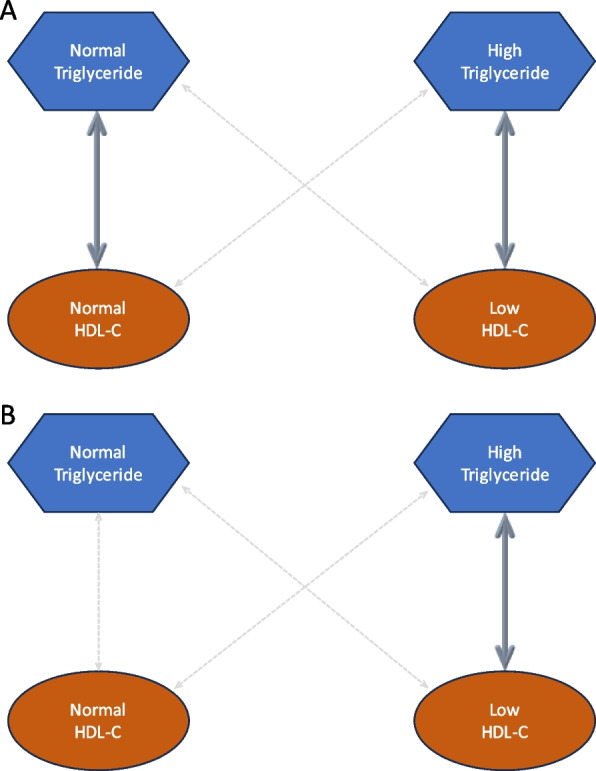
Correlation between TG levels and HDL-C levels in the studied groups with T2D./ **A** reveals the correlation between TG and HDL-C levels in females. There is a significant negative correlation between TG levels and HDL-C values in the group with high TG levels (150 mg/dL ≤ TG < 400 mg/dL) and low HDL-C concentrations ( HDL-C < 50 mg/ dL) and also in the group with normal TG (TG < 150 mg/dL) and normal HDL-C levels (50 mg/dL ≤ HDL-C) (marked by solid arrow). No significant correlation is investigated between TG levels and HDL-C levels in the group with high TG and normal HDL-C levels or the group with normal TG and low HDL-C levels (marked by dashed arrows). **B** depicts the correlation between TG and HDL-C levels in males. There is a significant negative correlation between TG levels and HDL-C levels in the group with high TG levels (150 mg/dL ≤ TG < 400 mg/ dL) and low HDL-C concentrations ( HDL-C < 40 mg/dL)(marked by solid arrow). No significant correlation is detected between TG levels and HDL-C levels in the remaining groups, including group with high TG and normal HDL-C levels (40 mg/dL ≤ HDL-C), group with normal TG (TG < 150 mg/dL) and low HDL-C levels, and group with normal TG and normal HDL-C levels (marked by dashed arrows). HDL-C: high-density lipoprotein cholesterol; TG: triglyceride; T2D: type 2 diabetes

A considerable inverse link was mentioned between concentrations of TG and HDL cholesterol in both categories of women and men with concurrent elevated TG range and low ranges of HDL-C (women: HDL-C < 50, men: HDL-C < 40) (R = - 0.008, women: *P* = 0.003 and men: *P* = 0.005). In contrast, a nonsignificant correlation was demonstrated in the category with concurrent high TG range and normal ranges of HDL-C (women: *P* = 0.521, men: *P* = 0.092). (Table 2). Moreover, a considerable inverse tie between HDL cholesterol and TG concentrations was assessed in females with normal amounts of HDL cholesterol and TG (R = -0.098, *P* = 0.006). However, the remaining groups had no significant association between TG and HDL cholesterol concentrations. (Table 2). Fig. 1 was designed to further clarify the connection between TG and HDL cholesterol amounts in the studied groups in women (Fig. 1(A)) and men (Fig. 1(B)).

**Table 2:** Correlation between TG values and HDL-C levels in the studied groups stratified by sex

Groups^@^	Female		Male	
	R	*P* value	R	*P* value
High TG & normal HDL-C^a^	-0.004	0.521	-0.009	0.092
High TG & low HDL-C^b^	-0.008	0.003^*^	-0.008	0.005^*^
Normal TG & normal HDL-C^c^	-0.098	0.006^*^	-0.042	0.208
Normal TG & low HDL-C^d^	-0.027	0.407	0.022	0.599

Dependent variable: HDL-C

Predictors: TG

*HDL-C* high-density lipoprotein cholesterol, *TG* triglyceride

@Group definition

^a^Group 1: patients with high TG (150 ≤ TG < 400 ) & normal HDL-C (40 ≤ HDL-C: male; 50 ≤ HDL-C: female)

^b^Group 2: patients with high TG (150 ≤ TG < 400 ) & low HDL-C (HDL-C < 40: male; HDL-C < 50: female)

^c^Group 3: patients with normal TG ( TG < 150 ) & normal HDL-C (40 ≤ HDL-C: male; 50 ≤ HDL-C: female)

^d^Group 4: patients with normal TG (TG < 150 ) & low HDL-C (HDL-C < 40: male; HDL-C < 50: female)

^*^Significant *P* value

### Comparison of clinical characteristics between groups with concurrent high TG and low HDL-C and groups with concurrent high TG and normal HDL-C

Due to the observed difference in the significance of the TG and HDL cholesterol association in the abovementioned categories, their demographic and clinical characteristics were also compared. In order to compare the characteristics of the category with a considerable association between TG and HDL cholesterol values with the group without a substantial correlation, they were matched by their gender, TG levels, and duration of diabetes, resulting in a total of 1036 patients in the category with high TG and decreased HDL-C, followed by 1070 matched participants in the other group. The mean ± (SD) has shown a substantially greater value in TG-to-HDL-C ratio and AIP in the category with concurrent low HDL cholesterol range and high ranges of TG. Age, VAI, hip circumference, SBP, DBP, history of hypertension, FBS, 2hPP, HbA1C, non-HDL, LDL-C, and total cholesterol have been presented with substantially higher mean ± (SD) values in groups with concurrent high TG range and normal ranges of HDL-C. The rest of the variables, including duration of diabetes, weight, height, BMI, WC, WHR, creatinine, urea, and eGFR, did not manifest a considerable difference between these two groups. Further data are provided in Table 3. A total of 77.06%, 14.45%, and 8.49% were receiving OAD, insulin, and their combination in the classification with decreased HDL cholesterol and high TG values. On the other hand, the use of OAD, insulin, and their combination in the studied population with elevated TG and normal HDL-C was as follows: 76.36%, 14.88%, and 8.76%, respectively, which was not significantly different from those with decreased HDL-C and elevated TG concentrations. Approximately 71.50% of persons with elevated TG and decreased HDL-C were taking atorvastatin, followed by rosuvastatin (28.50%). Moreover, the majority of the category with in-range HDL-C and high TG were receiving atorvastatin (71.70%), and the remaining were taking rosuvastatin (28.30%), which was not substantially different from those with elevated TG and decreased HDL-C concentrations.

**Table 3:** Comparison of demographic characteristics and laboratory tests between the group with high TG and low HDL-C and the group with high TG and normal HDL-C levels (matched by their gender, TG levels, and diabetes duration)

Variables				*P* value
		High TG with low HDL-C(*N* = 1036)	High TG with normal HDL-C (*N* = 1070)	
Duration of diabetes (yr)		8.42 ± 6.41^#^	8.54 ± 5.97	0.669
Age (yr)		54.41 ± 10.07	57.14 ± 10.14	< 0.001^*^
Female sex (%) (n)		50.7 (525)	50.50 (540)	0.924
Height (cm)		163.55 ± 9.27	163.26 ± 9.35	0.475
Weight (kg)		77.59 ± 12.59	78.45 ± 13.13	0.128
BMI (kg/m^2)		29.21 ± 4.16	29.45 ± 4.45	0.202
VAI		19.84 ± 3.71	20.27 ± 3.98	0.035^*^
Waist (cm)		98.46 ± 9.39	99.16 ± 10.00	0.098
Hip (cm)		105.34 ± 7.91	106.57 ± 8.59	0.004^*^
WHR		0.94 ± 0.05	0.94 ± 0.05	0.660
Blood pressure (mmHg)	Systolic	127.07 ± 15.71	131.64 ± 17.70	< 0.001^*^
	Diastolic	78.23 ± 8.38	79.96 ± 9.40	< 0.001^*^
FBS (mg/ dL)		166.06 ± 57.74	175.09 ± 62.25	0.001^*^
2hPP(mg/ dL)		231.38 ± 92.14	242.07 ± 93.98	0.008^*^
HbA1C (mg/ dL)		7.82 ± 1.71	8.11 ± 1.79	< 0.001^*^
Cholesterol (mg/ dL)		182.37 ± 40.08	205.50 ± 43.71	< 0.001^*^
HDL-C (mg/ dL)		36.99 ± 5.89	52.45 ± 9.02	< 0.001^*^
LDL-C (mg/ dL)		104.09 ± 34.64	113.68 ± 37.16	< 0.001^*^
TG (mg/ dL)		213.12 ± 48.13	212.44 ± 55.11	0.762
Non-HDL (mg/ dL)		145.38 ± 38.49	153.06 ± 42.76	< 0.001^*^
TG/HDL ratio		5.93 ± 1.82	4.17 ± 1.34	< 0.001^*^
AIP		1.74 ± 0.27	1.38 ± 0.30	< 0.001^*^
Creatinine (mg/ dL)		1.00 ± 0.22	1.00 ± 20	0.333
Urea (mg/ dL)		31.38 ± 10.77	32.07 ± 11.33	0.420
GFR		87.83 ± 24.32	86.55 ± 24.51	0.231
History of hypertension (%)		18.5 (192)	44.1 (472)	< 0.001^*^

*BMI* body mass index, *WHR* waist-to-hip ratio, *HbA1C* hemoglobin A1C, *FBS* fasting blood sugar, *2hPP* two-hour postprandial glucose, *GFR* glomerular filtration rate, *TG* triglyceride, *LDL-C* low-density lipoprotein cholesterol , *HDL-C* high-density lipoprotein cholesterol, *AIP* atherogenic index of plasma, *VAI* visceral adiposity index

^*^Significant *P* value

^#^Data are mean ± SD

### Association of TG concentrations with normal concentrations of HDL-C

In unadjusted analysis, significant relationships were investigated in having concurrent high TG (compared to normal TG) and normal HDL-C concentrations in combined (odds ratio [OR]: 0.559, 95% confidence intervals [CI]: 0.505– 0.618, *P* = < 0.001), men (OR: 0.574, CI: 0.491– 0.671, *P* = < 0.001), and women (OR: 0.574, CI: 0.500 – 0.658, *P* = < 0.001). Adjustments for duration of diabetes group, gender, age group, BMI group, DBP, SBP, WHR, HbA1C, and creatinine did not alter the significance of the mentioned results. (Table 4).

**Table 4:** Odds of having normal HDL-C levels in patients with high TG vs normal TG levels

		Normal levels of HDL-C		
		OR	95 CI%	*P* value
**Unadjusted**				
**TG categories**				
Combined	normal TG^1^	1.00 (reference)		
	high TG^1^	0.559	0.505– 0.618	< 0.001^*^
Men with	normal TG	1.00 (reference)		
	high TG	0.574	0.491– 0.671	< 0.00^*^
Women with	normal TG	1.00 (reference)		
	high TG	0.574	0.500– 0.658	< 0.001^*^
**Model 1**^a^				
Combined		0.582	0.525– 0.645	< 0.001^*^
Men		0.597	0.510– 0.700	< 0.001^*^
Women		0.585	0.510– 0.672	< 0.001^*^
**Model 2**^b^				
Combined		0.510	0.449 – 0.579	< 0.001^*^
Men		0.506	0.416 – 0.616	< 0.001^*^
Women		0.543	0.457– 0.644	< 0.001^*^

*OR* odds ratio, *95% CI* 95% confidence interval, *TG* triglyceride, *HDL-C* high-density lipoprotein cholesterol

^*^Significant *P* value

^a^Model 1 was adjusted for age group, sex, duration of diabetes group, and body mass index (BMI) group (age cutoff: 65 yrs., duration of diabetes cutoff: 5 yrs., BMI cutoff: 25 (kg/m^2))

^b^Model 2 was adjusted for age group, gender, duration of diabetes group, BMI group, systolic and diastolic blood pressure, waist/hip ratio (WHR), hemoglobin A1C (HbA1C), and creatinine (age cutoff: 65 yrs., duration of diabetes cutoff: 5 yrs., BMI cutoff: 25 (kg/m^2))

**Fig. 2:**
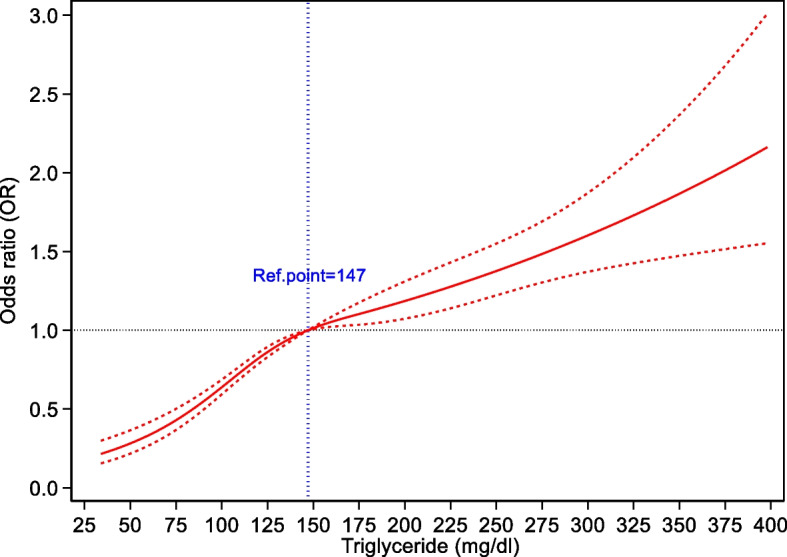
Association between TG and low HDL-C levels, allowing for nonlinear effects, with 95% CIs./ The RCS function was designed with 4 knots according to TG distribution. Curves show ORs compared with the chosen reference TG level of 147 mg/ dL. The ORs and 95% CIs of the RCS model with four knots for TG were 0.39 (CI 0.33–0.47) for 70 mg/ dL, 0.83 (CI 0.80–0.87) for 121 mg/ dL, 1.11 (CI 1.04–1.18) for 176 mg/ dL, and 1.62 (CI 1.38–1.91) for 304 mg/ dL. HDL-C: high-density lipoprotein cholesterol; TG: triglyceride; RCS: restricted cubic spline; OR: odds ratio; CI: confidence interval

The RCS in Fig. 2 demonstrates the connection between TG values and low HDL-C concentrations. A nonlinear association (all *P* < 0.001) between TG levels and low amounts of HDL cholesterol was detected. With a TG of 147 mg/ dL used as the reference (OR = 1), the ORs increased from 0.22 (CI 0.16–0.30) to 2.16 (CI 1.55–3.01) in the TG range of 34–398 mg/ dL. The ORs and 95% CIs of the RCS model with four knots for TG were 0.39 (CI 0.33–0.47) for 70 mg/ dL, 0.83 (CI 0.80–0.87) for 121 mg/ dL, 1.11 (CI 1.04–1.18) for 176 mg/ dL, and 1.62 (CI 1.38–1.91) for 304 mg/ dL.

**Fig. 3:**
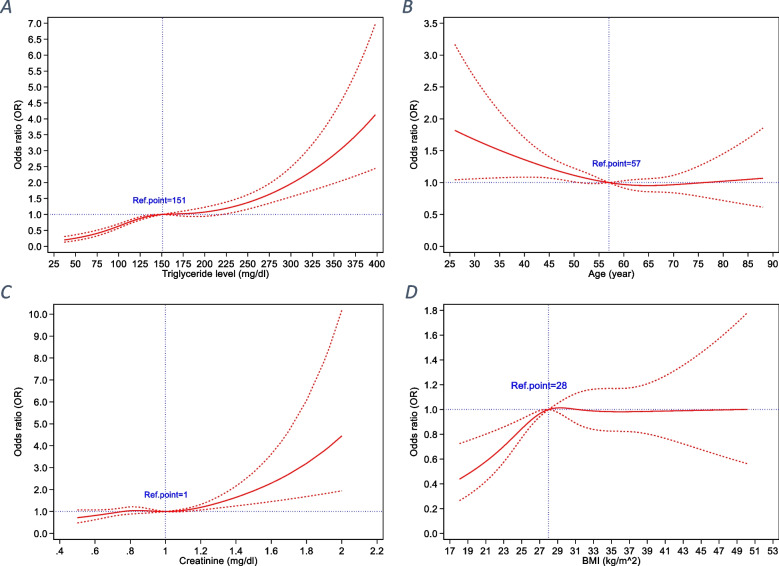
Association between TG (**A**), age (**B**), creatinine (**C**), and BMI (**D**) and low HDL-C levels in females adjusted for age, allowing for nonlinear effects, with 95% CIs. / The RCS function was designed with 4 knots according to TG (**A**), age (**B**), creatinine (**C**), and BMI (**D**) distribution. Curves show ORs compared with the chosen reference **A** TG level of 151 mg/ dL, **B** age of 57 years, **C** creatinine level of 1 mg/ dL, **D** BMI of 28 kg/m^2^. BMI: body mass index; HDL-C: high-density lipoprotein cholesterol; TG: triglyceride; RCS: restricted cubic spline; OR: odds ratio; CI: confidence interval

The RCS function in Fig. 3 indicates the relationship between TG, age, creatinine, and BMI and low HDL-C levels in females adjusted for age. The reference values for the mentioned curves were as follows: TG value of 151 mg/ dL, age of 57 years, creatinine value of 1 mg/ dL, and BMI of 28 kg/m2.

**Fig. 4:**
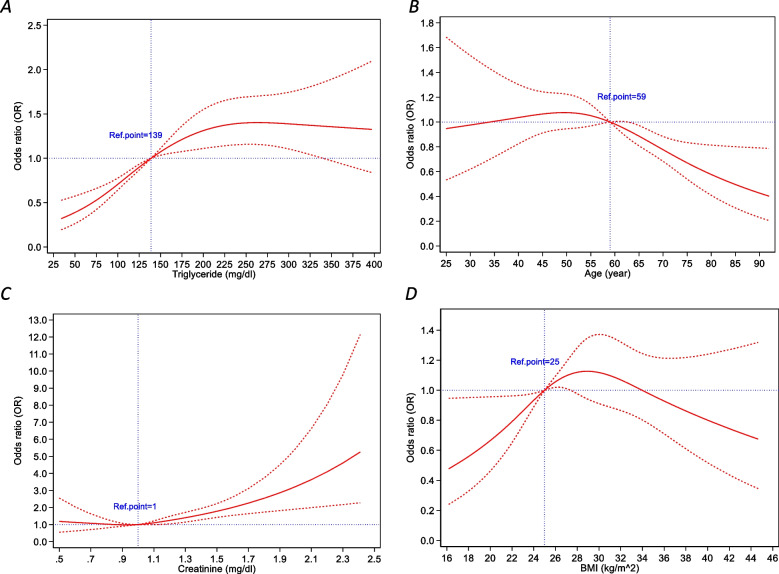
Association between TG (**A**), age (**B**), creatinine (**C**), and BMI (**D**) and low HDL-C levels in males adjusted for age, allowing for nonlinear effects, with 95% CIs. / The RCS function was designed with 4 knots according to TG (**A**), age (**B**), creatinine (**C**), and BMI (**D**) distribution. Curves show ORs compared with the chosen reference **A** TG level of 139 mg/ dL, **B** age of 59 years, **C** creatinine level of 1 mg/ dL, **D** BMI of 25 kg/m^2^. BMI: body mass index; HDL-C: high-density lipoprotein cholesterol; TG: triglyceride; RCS: restricted cubic spline; OR: odds ratio; CI: confidence interval

Moreover, the RCS function in Fig. 4 shows the relationship between TG, age, creatinine, and BMI and low HDL-C levels in males adjusted for age. The reference values for the mentioned curves were as follows: TG value of 139 mg/ dL, age of 59 years, creatinine value of 1 mg/ dL, and BMI of 25 kg/m2.

### Association of studied parameters with having normal HDL-C values

In the univariate logistic regression model, there were substantial positive relationships between HDL-C amounts and old age (65 years ≤ age) and height. In contrast, female sex and 25 kg/m^2^ ≤ BMI were inversely correlated with HDL-C levels. (Table 5). No considerable associations were illustrated between having normal HDL-C and the remaining variables.

**Table 5:** Univariate and multivariate analyses demonstrating odds of having normal HDL-C levels, according to studied parameters

Variables	OR	95 CI%	*P* value
**Univariate**			
Old age^a^	1.259	1.127–1.407	< 0.001^*^
Female sex	0.512	0.463– 0.566	< 0.001^*^
Height	1.210	1.160–1.270	< 0.001^*^
Overweight and obesity^b^	0.667	0.589– 0.754	< 0.001^*^
High HbA1C^c^	1.040	0.919–1.176	0.536
Creatinine	1.068	0.857–1.330	0.560
Systolic blood pressure	1.000	0.997–1.003	0.836
Diastolic blood pressure	1.001	0.996 –1.007	0.684
**Multivariate model**^d^			
Older age vs younger age	1.260	1.124–1.413	< 0.001^*^
Female sex vs male sex	0.467	0.416–0.523	< 0.001^*^
Creatinine (mg/ dL)	0.481	0.372–0.621	< 0.001^*^
Overweight and obesity vs normal and underweight BMI	0.786	0.691–0.894	< 0.001^*^

*OR* odds ratio, *95% CI* 95% confidence interval, *BMI* body mass index, *HbA1C* hemoglobin A1C

^*^Significant *P* value

^a^old age: 65 years ≤ age

^b^overweight and obesity: 25 kg/m^2^ ≤ BMI

^c^high HbA1C: 6.5% ≤ HbA1C

^d^The following variables were entered in the first step of multivariate logistic regression analysis: age group, sex, BMI group, HbA1c group, and creatinine. After excluding the variables with nonsignificant *P* values (greater than 0.05), age group, sex, and creatinine remained in the final multivariate model

By controlling for other variables in the multivariate model, age more than 65 years (OR 1.260, CI 1.124–1.413, *P* = < 0.001) was substantially correlated with having normal HDL-C values, while female sex (OR 0.467, CI 0.416–0.523, *P* = < 0.001), BMI over 25 kg/m^2^ (OR 0.786, CI 0.691–0.894, *P* = < 0.001), and higher serum levels of creatinine (OR 0.481, CI 0.372–0.621, *P* = < 0.001) remained substantial determinants against having normal levels of HDL-C. (Table 5).

The RCS models were implemented to establish the connection between age, creatinine, and BMI levels and having low values of HDL cholesterol (Fig. 3 in women and Fig. 4 in men). Each RCS had four knots with regard to the distribution of the associated variable.

## Discussion

According to the outcomes of the present investigation, high TG values were associated with a reduced likelihood of exhibiting normal HDL-C values. Patients who followed the rule of a considerable inverse association between HDL-C and TG were younger and had lower VAI, hip circumference, blood pressure, and glucose indices. However, there was no difference in other parameters, including weight, height, WC, WHR, and kidney function tests, between the two aforementioned categories. The strength of the link between triglyceride and HDL-C amounts was different in those with concurrent elevated TG and normal HDL cholesterol in comparison with individuals with concurrent decreased HDL-C and elevated TG. Significance disappeared in both sexes when the HDL-C levels reached the normal range. Furthermore, the odds of having normal HDL-C were augmented with increasing age and height, whereas they were lower with increasing BMI and female sex. However, according to the multivariate analysis, age, sex, BMI, and creatinine remained determinants of the likelihood of having normal levels of HDL-C.

The inverse correlation of TG and HDL-C values has long been a subject of debate. Miller et al. showed that each 50 mg/ dL decrease in the levels of triglyceride leads to a 0.5 mg/ dL rise in HDL cholesterol levels in patients with 200 ≤ TG mg/ dL and a 1.7 mg/ dL rise in the concentrations of HDL cholesterol in those with TG < 200 mg/ dL [14]. Consistent results were obtained in the current survey, which revealed that individuals with TG levels between 150 and 400 were 0.55 times less likely to have normal HDL-C values than individuals with normal TG levels. André J et al. stated that the reduction in HDL cholesterol amounts is greater than the decrease in HDL-apolipoprotein A-I levels by raising the plasma concentration of TG [15]. This could be due to the role of CETP in exchanging core lipids between VLDL and HDL [7]. Individuals with high TG levels had three times faster cholesterol ester transfer to VLDL compared with normal TG levels [15]. Moreover, by raising TG levels, the impaired catabolism of TG-containing lipoprotein leads to inadequate transfer of surface factors to nascent HDL particles [14]. lecithin-cholesterol acyltransferase (LCAT) is a factor needed for cholesterol ester production. Therefore, a reduction in LCAT activity could result in lower values of HDL-C [29]. As a result, LCAT and CETP could be regulating determinants of HDL-C levels [15]. Another explanation for reducing HDL-C levels with higher values of TG ​​is the disappearance of the positive correlation between CETP and LCAT by increasing TG levels [30].

Females were 0.46 times less likely to have normal HDL-C levels than males. Therefore, female sex was correlated with having reduced HDL-C levels. The current findings add to the evidence obtained in Yang’s national cross-sectional study, where low HDL-C was 2.890 times more prevalent in women than in men. This observation might be due to the different lifestyles, behaviors, stress levels, and nutrition intake in women [31]. Social determinants of health in females may contribute to their lower HDL-C levels [32, 33].

The present survey found that HbA1C, 2hPP, and FBS were lower in those with a substantial inverse association of HDL-C and TG concentrations. Prior studies were controversial. Huang et al. reported that HbA1c manifested an inverse correlation with HDL-C in patients with diabetes [34]. However, others showed no substantial link between HDL-C concentrations and the serum concentrations of HbA1C, 2Hpp, or FBS [35, 36]. From a biochemistry view, impaired blood glucose results in insulin-resistant fat cells releasing more free fatty acids (FFAs). Consequently, TG production is then enhanced by increased FFAs. As a result, diabetes control is essential to control the lipid profile [37].

The odds of having normal HDL-C levels decreased with increased creatinine values in this experiment. This is in line with expectations. You A. et al. reported that eGFR values below 90 ml/min/1.73 m2 had a substantial association with low HDL-C levels [38]. Another study further elucidated the positive link between HDL-C concentrations and eGFR levels [39]. This event may be ascribed to a combination of impaired apolipoprotein A-I production and increased catabolism, as well as reduced appetite and subsequent malnutrition in the subjects with CKD [38, 39]. This observation supports the idea that reducing creatinine levels may play a role in HDL-C control.

The current investigation revealed that patients with 25 kg/m2 ≤ BMI were 0.78 times less likely to have normal HDL-C levels than those with BMI < 25 kg/m2 in this survey. The present observations are in accordance with previously published studies in which HDL-C values had a substantial inverse connection with BMI [40, 41]. There were several explanations for the negative effect of BMI on HDL-C amounts. Obesity can stimulate adipocytes to increase HDL_2_ uptake and provoke apolipoprotein A-I catabolism on HDL particles [42]. Therefore, a reduction in BMI may help control lipid profiles. Hip and waist circumference, as markers of peripheral adiposity, were also shown to have a relationship with HDL-C levels. Seidell et al. noted that Low HDL cholesterol was correlated with a slim hip circumference [43]. The current survey results also manifested that hip circumferences had lower values in persons with elevated TG and decreased HDL-C values. This could be explained due to the reduced subcutaneous fat in narrow hip circumference [43].

Harman et al. revealed a considerable positive relationship between age and HDL- levels [44]. Similarly, a study in China showed that HDL-C amounts increased with age in both hypertensive and normotensive individuals [45]. The present study also confirmed that older individuals (65 years ≤ age) were 1.26 times more likely to have normal HDL-C levels than younger individuals. Additionally, age was lower in those with a substantial inverse association of HDL-C and TG concentrations. The observed association between aging and enhancement of HDL-C concentrations can be due to increased survival of those with higher levels of HDL-C in cross-sectional studies. On the other hand, a cohort study demonstrated no significant link between HDL-C levels and advancing age [46]. Prospective investigations have usually indicated that HDL-C is reduced or sustained with aging. Walter et al. explained that substituting apolipoprotein A-I for the acute-phase reactant serum amyloid A and the telomere-driven senescence mechanism based on the loss of divisional capacity can clarify these results in prospective studies [47].

The current survey manifested that increasing height was connected with an enhanced likelihood of having normal HDL-C levels. This is consistent with Kouda’s results that an inverse link was detected between HDL-C values and height [48]. However, another survey showed a considerable positive association between height and HDL-C concentrations [49]. Although the exact mechanism supporting this association is yet unclear, hormonal pathways could contribute to this link. Growth hormone can increase lipid metabolism through lipolytic action. Thyroid hormone is also implicated in the metabolism of cholesterol and fatty acids. Additionally, osteocalcin results in increased HDL-C levels through enhanced expression of the adiponectin gene [49].

The current analysis provides evidence that systolic and diastolic blood pressure values were lower in the category with concurrent reduced HDL cholesterol and raised TG values compared to the other group. Although the connection between blood pressure and HDL-C amounts has been a subject of controversy in prior experiments, a number of investigations established a positive link between HDL-C levels and higher blood pressure[50, 51]. However, this abovementioned relation was not detected in all individuals. Yang et al. showed that controlling for BMI further converted the inverse correlation between HDL-C and blood pressure to a positive relationship, but this only applied to men [50]. Moreover, Shimizu et al. noticed that patients without CD34-positive cell levels did not manifest a considerable link between blood pressure and HDL-C values [51]. The precise relationship between HDL-C levels and blood pressure has yet to be well studied.

### Study strengths and limitations

To the extent of the authors’ knowledge, this is the first time that the inverse interrelationship of HDL-C and TG has been assessed and the first time that the demographic features of groups in which the correlation between TG and HDL-C is significant have been investigated. This survey had a relatively large sample size. Moreover, various covariates, such as demographic and anthropometric factors and laboratory data, were incorporated into the statistical analyses.

Due to the cross-sectional nature of this survey, the salutary clinical impact of these results cannot be addressed, and discerning whether the relationship mentioned between HDL-C and these variants is a true effect, a survivor effect, or a cohort effect is not possible. Therefore, it is of great value to conduct cohorts to follow the trajectory of HDL-C levels in each patient with different valuables. Several variables were assessed at a single time point, which could negatively affect the accuracy of the data.

## Conclusion

Considering the challenges in boosting HDL-C levels, it may be possible to develop more effective treatments by understanding the factors that link to HDL-C levels. HDL-C manifested an inverse correlation with TG levels in a particular group of patients. Both men and women with decreased HDL-C and elevated TG concentrations exhibited a connection between their HDL-C and TG values. Many factors contributed to this association, including age, hip circumference, VAI, blood pressure, FBS, 2hPP, HbA1C, and lipid parameters. The likelihood of an inverse association between HDL cholesterol and TG values was higher in younger individuals with lower hip circumference, VAI, and well-controlled blood pressure and glucose indices. Other demographic and laboratory findings were also found to be connected with HDL-C values. Age younger than 65 years, female sex, BMI over 25 kg/m^2^, and higher creatinine levels were associated with having lower amounts of HDL-C.

## Data Availability

The datasets used and/or analyzed during the current study are available from the corresponding author on reasonable request.

## Abbreviations

ADA: The American Diabetes Association
AIP: Atherogenic index of plasma
BMI: Body mass index
CETP: Cholesterol ester transfer protein
CKD: Chronic kidney disease
DBP: Diastolic blood pressure
eGFR: Estimated glomerular filtration rate
FBS: Fasting blood sugar
FFAs: Free fatty acids
HbA1C: Hemoglobin A1C
HDL-C: High-density lipoprotein cholesterol
HPLC: High-performance liquid chromatography
LCAT: Lecithin-cholesterol acyltransferase
LDL-C: Low-density lipoprotein cholesterol
NCEP ATP III: National Cholesterol Education Program-Adult Treatment Panel III
OAD: Oral anti-diabetic agents
RCS: Restricted cubic spline
SBP: Systolic blood pressure
TG: Triglyceride
T2D: Type 2 diabetes
VAI: Visceral adiposity index
VLDL: Very low-density lipoprotein
WC: Waist circumference
WHR: Waist-to-hip ratio
